# Prevention of Recurrent Childhood Caries with Probiotic Supplements: A Randomized Controlled Trial with a 12-Month Follow-Up

**DOI:** 10.1007/s12602-022-09913-9

**Published:** 2022-01-26

**Authors:** P. Hasslöf, L. Granqvist, C. Stecksén-Blicks, S. Twetman

**Affiliations:** 1 Department of Odontology, Pediatric Dentistry, Faculty of Medicine, Umeå University, 90187 Umeå, Sweden; 2 Pediatric Dentistry, Eastman Dental Institute, Stockholm, Sweden; 3 Department of Odontology, Faculty of Health and Medical Sciences, University of Copenhagen, Copenhagen, Denmark

**Keywords:** Dental caries, *Limosilactobacillus*, Preschool children, Probiotic drops

## Abstract

The aim of this study was to evaluate the effect of drops containing probiotic bacteria on the recurrence of dental caries in preschool children. The study employed a randomized, placebo-controlled, double-blinded design with two parallel arms. 38 preschool children were enrolled after comprehensive restorative treatment under general anesthesia or conscious sedation (baseline), and they were followed up after 6 and 12 months. Parents of children in the test group were instructed to give 5 daily drops containing two strains of *Limosilactobacillus reuteri* (DSM 17938 and ATCC PTA 5289) at bedtime. The placebo drops were identically composed but lacked bacteria. The duration of the intervention was 12 months. The primary endpoint was recurrence of new caries lesions on subject level (yes/no), and secondary endpoints were presence of dental plaque and gingivitis. We found high rate of recurrent moderate and extensive lesions after 12 months (67%) but there were no significant differences between the groups. We observed no beneficial effects on dental plaque or gingival inflammation. The findings were however uncertain and inconclusive due to lack of power, a consequence of the COVID-19 pandemic. ClinTrials.gov Identifier: (NCT04929340), June 18, 2021; retrospectively registered.

## Introduction

Early childhood caries (ECC) is a common disease associated with impaired oral health-related quality of life for the child and high costs for families and society [1]. The etiology is complex with numerous biological, medical, behavioral, psychological, cultural, and lifestyle factors linked to its onset. It is commonly postulated that ECC is a preventable disease, but systematic reviews have shown that preventive measures are only partly successful [2, 3]. Unfortunately, also the tertiary prevention and management of ECC relies on evidence of low quality and the 12-month relapse rate in terms of recurrent caries after conservative treatment is reported to range from 20 to 80% [4–8]. Consequently, along with mandatory fluoride exposure and sugar restrictions, there is a need to develop novel strategies to combat the development of new caries lesions in children with a history of ECC.

Probiotic bacteria are “live microorganisms which, when administered in adequate amounts, confer a health benefit on the host” [9]. The background thinking is that a harmless effector strain implanted in the host’s microflora can maintain or restore a natural microbiome by interference and/or inhibition of other microorganisms. In this context, the microbial colonization and maturation of the oral cavity early in life is of particular interest since the first 1000 days of life provide a window of opportunity for modulating the microbiota through interventions with pre- and probiotics to promote a healthy growth and development [10]. Several previous studies have indicated that infants, toddlers, and preschool children that are exposed to probiotic supplements can display significant reductions in caries incidence in the primary dentition [11, 12]. We therefore thought it was of interest to evaluate if the use of probiotic supplements as adjunct to standard care after restorative treatment could reduce the risk of recurrent decay. The aim of this study was to compare the effect of drops containing probiotic bacteria on the incidence of dental caries with placebo drops in preschool children after a comprehensive restorative treatment under general anesthesia or conscious sedation. The null hypothesis was that there would be no significant difference between the two interventions.

## Material and Methods

### Study Design

The project employed a randomized, placebo-controlled double-blind design with two parallel arms. The setting was three specialist pediatric dentistry clinics: the Maxillofacial Unit, Halland Hospital, Halmstad, Sweden; Eastman Dental Institute, Stockholm, Sweden; and Kronan, Stockholm, Sweden. We enrolled preschool children referred for restorative treatment and extractions under general anesthesia or conscious sedation. After informed parental consent, children were randomly assigned to either a test or placebo group. The duration of the intervention was 12 months, and the children were recalled after 6 and 12 months for follow-up examinations. The study protocol gained approval from the Regional Ethical Board for medical research, Umeå, Sweden (Dnr 2017–20-31 M), and was registered in ClinTrials.gov (NCT04929340).

### Study Group

We consecutively recruited preschool children, 2–5 years of age, diagnosed with ECC or severe ECC. The children were referred from their ordinary dental team to the specialist clinics for treatment due to low age, cooperation problems, or extensive treatment needs. After the operative care was completed, we informed the parents on the purpose of the project and invited them to enroll with their child. All children were potentially eligible, but we excluded (a) medically comprised children, (b) children with severe cognitive problems or dysfunctional behavior, (c) children with refugee status, and (d) children in families planning to relocate within the next year.

### Intervention

After written consent from both parents, we allocated the children randomly to the test or the placebo group with the aid of a computer-generated random binary list. After receiving parental consent, an envelope was opened which gave each child an equal 50% chance to be allocated to one of the groups. Parents were instructed to give their child 5 drops each day before bedtime but after tooth brushing. The active drops contained two strains of the probiotic bacteria *Limosilactobacillus reuteri* (DSM 17938 and ATCC PTA 5289) with a minimum of 100 million live bacteria of each strain. The placebo drops had an identical composition, color, and taste (orange flavor) but contained no probiotic bacteria. The parents were instructed to shake the bottles well before use. The test and the placebo drops were prepared and provided by BioGaia AB (Stockholm Sweden) in color-coded bottles (yellow and blue) to cover a 6-month use. We distributed a new set of bottles at the 6-month follow-up. The standard preventive care after the baseline treatment was as follows: all parents received information about caries etiology, dietary advice with focus on limiting consumption of sugar-containing food and beverages, and oral hygiene instruction. Children considered at very high caries risk were recalled to a dental assistant for positive reinforcement every third month. In addition, we supplied all children with a toothbrush and standard sodium fluoride toothpaste (1100 ppm F). The parents were strongly encouraged to brush their child’s teeth twice daily.

### Clinical Procedures

We extracted data on caries prevalence (decayed, extracted, and filled teeth/surfaces; deft/defs) from the dental records. Data on the family characteristics, dietary habits, and oral hygiene routines were collected with a questionnaire. Five experienced and calibrated pediatric dentists performed the conservative treatment under general anesthesia or conscious sedation and the number of fillings and extracted teeth was registered. The same dentist performed the baseline and recall examinations. Caries was scored according to the modified ICDAS II criteria [13] as follows: ICDAS 0 = sound, ICDAS 1–2 = initial lesion, ICDAS 3–4 = moderate lesion, and ICDAS 5–6 = extensive lesion. The gingival condition was expressed as “bleeding-on-brushing”; the teeth were gently brushed with a disposable toothbrush and any bleeding along the gingival margin that appeared within 30 s was scored as “yes.” Likewise, the presence of visible supra-gingival plaque on the buccal surfaces of the upper anterior teeth (if available) was registered as “yes.” The primary endpoint was recurrence of new caries lesions on subject level, dichotomized as “yes” or “no.” Secondary endpoints were presence of gingivitis and visible dental plaque. We encouraged the compliance with the study protocol through regular telephone contacts and the parents were asked to bring back the non-used bottles to the clinic. We informed the child’s regular dentist on the purpose of the study and this dentist kept the responsibility for any treatment decisions during the follow-up period. We instructed parents to report any possible or perceived adverse event to the principal investigator.

### Statistical Methods

The data was processed by the IBM SPSS Statistics 27 software. Chi-square tests were used for categorical data and proportions and for continuous data, the Wilcoxon test was applied. We expressed caries recurrence on subject level as relative risk (RR) and 95% confidence interval. A *p* value < 0.05 was considered statistically significant. The study was blind for the children, parents, clinicians, and researchers and the group allocation was kept concealed by an independent monitor at Umeå University. The code was not broken until after the statistical calculations.

### Power Calculation

We used data on caries relapse after treatment (40%) among preschool children published by Berkowitz and coworkers [6]. We assumed that a 50% difference between the study groups would be clinically relevant and important. Thus, 70 children in each arm should be enrolled to provide sufficient power with *α* (probability of a Type I error) set at 5% and *β* (probability of type II error) set at 80%, and this would allow an expected 10% dropout rate.

## Results

**Fig. 1:**
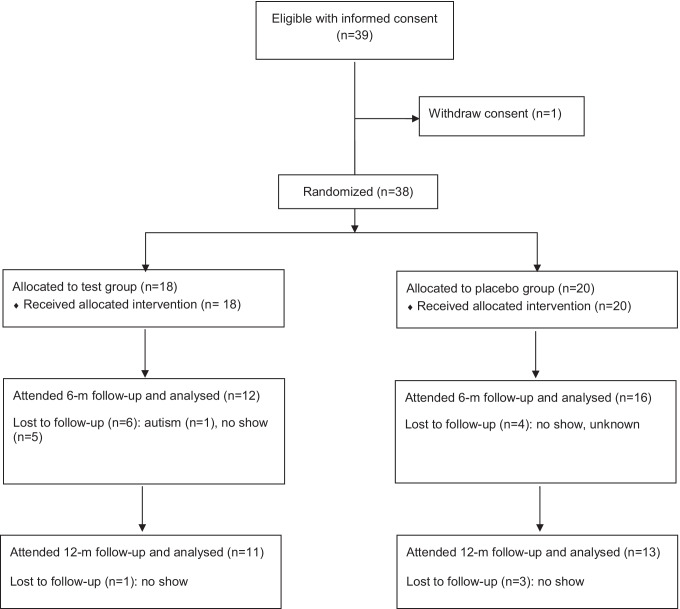
Flow chart of the trial

The consecutive enrollment of patients started January 2017 but the process was discontinued in March 2020 as the hospital’s resources, in particular the anesthesiologists, had to focus on the COVID-19 care. Moreover, non-emergency dental visits were rescheduled and/or postponed. At that time, 38 children had started the intervention but only 28 and 24 children were able to show up after 6 and 12 months, respectively. Thus, the ongoing pandemic forced us to terminate the project. The detailed flowchart is showed in Fig. 1.

The baseline characteristics is presented in Table 1. There were no significant differences between the groups regarding age, sex, deft, defs, oral hygiene routines, or sugar intake. A higher mean number of extractions at the baseline treatment was noted in the placebo group compared to the test group (4.2 vs 2.0 teeth), but this difference was not statistically significant. Ninety-eight percent of the participants used fluoride toothpaste regularly and 62% of the parents brushed their children’s teeth twice a day (data not shown).

**Table 1:** Baseline characteristics and performed conservative treatment prior to intervention

	Test (*n* = 18)	Placebo (*n* = 20)	*p*^a^
Age, mean, range (months)	41.5 (10.8); 22–58	41.9 (12.5); 24–62	NS
Girls/boys (*n*)	7/11	11/9	NS
deft, mean (SD); range	9.9 (4.6); 2–19	9.5 (4.0); 1–18	NS
defs, mean (SD); range	16.9 (8.4); 4–37	15.6 (9.7); 2–34	NS
Siblings, > 1 (*n*)	3 (17%)	7 (35%)	NS
Tooth brushing by parents, ≥ daily (*n*)	13 (72%)	17 (85%)	NS
Fluoride toothpaste, yes (*n*)	18 (100%)	19 (95%)	NS
Nocturnal breastfeeding, yes (*n*)	4 (22%)	1 (5%)	NS
Cookies, buns, ≥ every week (*n*)	13 (72%)	15 (75%)	NS
Sweet drinks, candy, ≥ every week (*n*)	13 (72%)	16 (80%)	NS
*Baseline conservative treatment:*			
Fillings, mean number (SD); range	3.0 (1.8); 0–6	3.3 (2.4): 1–7	NS
Extractions, mean number (SD); range	2.0 (2.0); 0–5	4.2 (2.6): 0–8	NS

^a^Values expressed as percentage were compared with a chi-squared test for comparison of proportions in independent samples; Mean values were subjected to two-sided Wilcoxon test

*NS* not statistically significant; *deft*, decayed, extracted, filled teeth; *defs*, decayed, extracted, filled tooth surfaces

The recurrence of moderate and extensive lesions (ICDAS 3–6) on subject level was 57% after 6 months and 67% after 12 months and again, there was no difference between the study groups (Table 2). The prevalence of bleeding-on-brushing was around 18% after 6 and 12 months in the test group and slightly lower in the placebo group (Table 3). Around 70% of the children in both groups had visible plaque on their upper anterior teeth after 12 months. No side effects or adverse events were reported during the course of the study.

**Table 2:** Recurrent caries (yes/no) at the 6- and 12-month follow-ups with calculated relative risk (RR) and 95% confidence interval

Time	Test	Placebo	RR (95% CI)	*p*
	Yes/No	Yes/No		
6 months	*n* = 12	*n* = 16		
ICDAS 1–2	7/5 (58%)	7/9 (48%)	1.33 (0.64; 2.77)	NS
ICDAS 3–6	8/4 (67%)	8/8 (50%)	1.33 (0.71; 2.51)	NS
12 months	*n* = 11	*n* = 13		
ICDAS 1–2	7/4 (64%)	6/7 (46%)	1.38 (0.66; 2.88)	NS
ICDAS 3–6	9/2 (75%)	7/6 (54%)	1.52 (0.85; 2.70)	NS

*ICDAS* International Caries Detection and Assessment System, see “Material and Methods” section for explanation

*NS* not statistically significant

**Table 3:** Visible plaque on the buccal surfaces of the anterior upper teeth and bleeding-on-brushing at baseline and follow-up

	Test	Placebo	*p*^a^
Visible plaque	Yes (%)	Yes (%)	
6 months	58%	31%	NS
12 months	73%	69%	NS
Bleeding-on-brushing			
6 months	17%	6%	NS
12 months	18%	8%	NS

^a^Chi-squared test for comparison of proportions (independent samples), expressed as percentage

*NS* not statistically significant

## Discussion

Due to the unforeseen circumstances caused by the pandemic, we decided to terminate the project but the obligation and responsibility to report the outcome of the study remains. The main finding was a high recurrence rate of early childhood caries in both groups, and we were therefore unable to reject the null hypothesis. The results must however be considered as highly unreliable. Firstly, the study failed to reach statistical power since only half of the intended study population was enrolled. Indeed, the high 12-month dropout rate (37%) made relevant comparisons even less possible. The high attrition rate was unexpected, but many children belonged to families with immigrant background that simply failed to show up for unknown reasons. Secondly, we lack reliable information on the compliance. It is possible that failure to comply with the study protocol, in spite of reminders, was one of the reasons for the frequent no-shows. The imbalance in number of extractions at the baseline dental treatment between the groups was a third factor that brought uncertainty to the outcome since a higher number of remaining tooth surfaces at risk for new caries lesions was present in the test group. A final shortcoming was that we were unable to perform a formal inter-examiner reliability evaluation for practical reasons.

The high caries recurrence rate reported here was disappointing, albeit in agreement with several previous trials [5, 6, 8]. It is however important to keep in mind that early childhood caries has a very complex etiology and that the recruited children displayed a high cariogenic challenge and disease activity. The conservative treatment with fillings and extractions certainly addressed the symptoms but we obviously failed to inform and motivate parents to take action and adopt the dietary and lifestyle-related changes needed to avoid the relapse. Daily use of fluoride toothpaste is crucial in caries prevention. At baseline, 98% of the caregivers reported regular use of fluoride toothpaste. However, only 62% helped their children with dental hygiene twice a day, as recommended. The standard preventive care delivered in connection with the baseline restorative care and afterwards was obviously not sufficient in this cohort as a majority of the children in both groups had visible plaque on their teeth at the 12-month follow-up. From an early age of the child, there is obviously room for improvements in parental involvement and engagement through a multidisciplinary skill mix based on previous knowledge gained from chronic disease management [14].

The probiotic supplements used in this study contained two *Limosilactobacillus* strains isolated from human breastmilk (DSM 17938) and the oral cavity (ATCC PTA 5289). This combination has previously been reported effective in the prevention and management of gingivitis and periodontitis in adults [15, 16], and to induce remineralization of initial caries lesions in schoolchildren [17]. Lozenges containing *L. reuteri* can also shift the composition of the oral biofilm and reduce the number of *S. mutans*, *S. sobrinus*, *P. gingivalis*, and *A. actinomycetemcomitans* in saliva in subjects under treatment of fixed orthodontic appliances [18]. In the abovementioned studies, the vehicles for administration were slow-melting lozenges but drops were chosen here to fit the low age of the study group and for parent convenience. In Scandinavia, many custodians are used to give their young children D-vitamin drops on a daily basis, and this mode was therefore thought to facilitate compliance. Probiotic supplements exert local antibacterial effects in dental biofilm as well as indirect effects via gut-mediated influences on the immune system [19]. Previous studies with one of the tested *L. reuteri* strains (DSM 17938) in drops have indicated that such intervention can reduce caries and bronchial inflammation in asthmatic children and alleviate infantile colic and IgE-associated eczema [20–23]. It is however possible that the contact time and retention of the probiotic strains to the biofilms in the oral environment were insufficient to overcome the cariogenic challenges and affect the caries development. To improve local co-aggregation and competitive exclusion, slowly melting lozenges could possibly be a better alternative for children over 3 years of age. It may also be a matter of dose response, which is an open question that requires further research. This investigation failed to evaluate effect of probiotic bacteria but raised the urgent need to improve the methods for communicating and implementing basic preventive care for children in with ECC.

## Conclusions

Administration of probiotic drops containing two strains of *L. reuteri* failed to reduce the recurrence of early childhood caries in comparison with a placebo group. The findings were however uncertain and inconclusive as the study was discontinued due to the COVID-19 pandemic, and thereby lacked sufficient power.

## Data Availability

The datasets generated during the current study are available from the corresponding author on reasonable request.
